# ID 194 - RWE by Chart Review: Challenges and Opportunities from a Retrospective Brazilian Experience

**DOI:** 10.5327/2237-9622.2025.v34s1.194

**Published:** 2025-11-25

**Authors:** Patricia Lopes de Almeida Simon, L. S. Piton, H. W. G. Santos, L. M. Leobaldo, F. Z. Nazareth, A. Jain, A. Guarin

**Keywords:** real-world data, real-world evidence, data quality

## Abstract

**Introdução:**

Randomized controlled trials (RCTs) are the gold standard for generating evidence. The data collection outside clinical research environment can add value to clinical unmet medical needs and shows the reality of patient's journey. Together, RCTs and Real-World Evidence (RWE) create a complementary framework for disease management. In the literature, there is enough evidence on RCT and RWE limitations, but few about challenges of collecting data in the RWE scenario to enhance study quality. This work shares experience, identify barriers and potential solutions to generate robust RWE based on chart review.

**Método:**

IRIS Brazil is an ongoing, retrospective, site-based study to review medical record of patients who have received Palbociclib combinations in first-line therapy for HER2-, HR+ metastatic breast cancer in Brazil private setting to assess clinical outcomes and treatment patterns. The data was collected using an online capture system managed by a contract research organization (CRO). Interviews were conducted with the CRO and Sponsor teams aimed to identify the main barriers and improvements for conducting the present RWE study.

**Resultados:**

Three main barriers were identified to obtaining data integrity: research institution qualification by a robust feasibility assessment, institution training and data quality check. Analyzing the data availability, team experience in RWE studies and overall site organization were the main parts of an upstanding research institution feasibility. In a qualified institution, the CRO effectively trains staff on study details and protocols, minimizing risks and enhancing study adherence. But, it was observed that only the initial training wasn’t enough to comply with all complexity of the sites accounted for RWE study. Lastly, the data quality check is an indispensable checkpoint in data entrance. Site monitoring and data management minimize inconsistencies, clarify protocol points, and reduce human error frequency.

**Conclusão:**

RWE studies comprehensively capture essential clinical outcomes related to patient therapeutic management outside the RCT-selected population. An additional advantage is accessing physician-exclusive information through expert judgment in a real-world setting. In conclusion, robust feasibility assessment, comprehensive site staff protocol training and quality data monitoring can help in keeping data integrity and generating high quality publications.

